# Top-Down Disconnectivity in Schizophrenia During P300 Tasks

**DOI:** 10.3389/fncom.2018.00033

**Published:** 2018-05-23

**Authors:** Fali Li, Jiuju Wang, Yuanling Jiang, Yajing Si, Wenjing Peng, Limeng Song, Yi Jiang, Yangsong Zhang, Wentian Dong, Dezhong Yao, Peng Xu

**Affiliations:** ^1^MOE Key Lab for Neuroinformation, The Clinical Hospital of Chengdu Brain Science Institute, University of Electronic Science and Technology of China, Chengdu, China; ^2^Institute of Mental Health, Peking University Sixth Hospital, National Clinical Research Center for Mental Disorders & Key Laboratory of Mental Health, Ministry of Health, Peking University, Beijing, China; ^3^School of Computer Science and Technology, Southwest University of Science and Technology, Mianyang, China; ^4^Center for Information in Medicine, School of Life Science and Technology, University of Electronic Science and Technology of China, Chengdu, China

**Keywords:** Dynamic Causal Modeling, P300, schizophrenia, top-down disconnectivity, compensatory mechanisms

## Abstract

Cognitive deficits in schizophrenia are correlated with the dysfunctions of distinct brain regions including anterior cingulate cortex (ACC) and prefrontal cortex (PFC). Apart from the dysfunctions of the intrinsic connectivity of related areas, how the coupled neural populations work is also crucial in related processes. Twenty-four patients with schizophrenia (SZs) and 24 matched healthy controls (HCs) were recruited in our study. Based on the electroencephalogram (EEG) datasets recorded, the Dynamic Causal Modeling (DCM) was then adopted to estimate how the brain architecture adapts among related areas in SZs and to investigate the mechanism that accounts for their cognitive deficits. The distinct winning models in SZs and HCs consistently emphasized the importance of ACC in regulating the elicitations of P300s. Specifically, comparing to that in HCs, the winning model in SZs uncovered a compensatory pathway from dorsolateral PFC to intraparietal sulcus that promised the SZs' accomplishing P300 tasks. The findings demonstrated that the “disconnectivity hypothesis” is helpful and useful in explaining the cognitive deficits in SZs, while the brain architecture adapted with related compensatory pathway promises the limited brain cognitions in SZs. This study provides a new viewpoint that deepens our understanding of the cognitive deficits in schizophrenia.

## Introduction

The dysfunctions of multiple brain regions disrupt the efficient information processing in patients with schizophrenia (SZs) (Insel, 2010); distinct studies have proved the SZs show the comparable abnormalities in multiple brain cognitions, such as attention and working memory, when compared to healthy controls (HCs) (Prado et al., 2011; Onitsuka et al., 2013; Smucny et al., 2013). Using neuroimaging techniques including electroencephalogram (EEG) and functional magnetic resonance imaging (fMRI), etc., brain regions, such as anterior cingulate cortex (ACC) and prefrontal cortex (PFC), are confirmed their abnormalities in SZs (Hulshoff Pol et al., 2002; Ohtani et al., 2014; Mouchlianitis et al., 2015). For example, in brain regions including thalamus, PFC, and a large posterior area centered on occipito-temporo-parietal junction, the reduced volume of gray matter is reported in SZs (Schuster et al., 2012). The dysfunctions of brain cognitions in SZs are usually interpreted as disturbed neural connectivity that is termed as the “disconnectivity hypothesis” (Stephan et al., 2009), i.e., the inter-regional disconnection in prefrontal-temporal (Ford et al., 2002) and cortico-cerebellar circuits (Wiser et al., 1998). For example, the fronto-parietal attention network covering brain regions, such as the higher visual area, intraparietal sulcus (IPS), and temporoparietal junction, etc., was proved to be dysfunctioned and further led to the cognitive deficits in SZs (Roiser et al., 2013), as well as the atypical top-down cognitive processes (Dima et al., 2010; Cook et al., 2012).

P300 is a physiological index of cognitive processes (Polich, 2007; Li et al., 2015). The elicitations of P300s are attributed to the simultaneous cooperation of distinct P300 generators (Bledowski et al., 2004; Molina et al., 2005). Once the dysfunctions of related areas happen, the deficits of P300s occur, i.e., the decreased amplitude and prolonged latency (Yamaguchi and Knight, 1991; Daffner et al., 2003). In fact, P300 has been regarded as the endophenotype of schizophrenia (Martin-Loeches et al., 2001; Bramon et al., 2005) and also used to index the neurobiological vulnerability in SZs. Multiple studies have proved the usefulness of the event-related potentials (ERPs) (i.e., P300 in this work) in evaluating the individual task behaviors, as well as in SZs (Leitman et al., 2010; Rissling et al., 2010; Pan et al., 2014). For example, the P300 amplitudes of SZs were significantly decreased along the midline electrodes and both bilateral temporal areas compared to that of HCs, as well as the decreased source activations (Kim et al., 2014). In addition, the P300 reduction was related to abnormal assessments of related social functioning and social personal adjustment in patients, and compared to the N100 or P200, the reduced P300 amplitudes were the best predictor for the psychosis in the ultra high risk group (Tricht et al., 2010). Therefore, identifying the P300 variability between SZs and HCs helps address the cognitive deficits in SZs (Jeon and Polich, 2003; Bramon et al., 2004; Fusar-Poli et al., 2011).

Our brain works on a large-scale complex network that distributes on large numbers of related brain regions (Petersen and Sporns, 2015); and within the network, the information is processed efficiently with the contributions from the spatially distributed but functionally linked regions (Sharma and Baron, 2013; Li et al., 2016). Using approaches that rely on the physiological models, such as Dynamic Causal Modeling (DCM) (Friston et al., 2003), the dynamic patterns among multiple specialized areas can be marked out and further adopted to account for the mechanism of brain functions (Brázdil et al., 2007; Liu et al., 2016), as well as the cognitive deficits in SZs (Dima et al., 2010; Wagner et al., 2015). For example, in DCM study conducted by Diez et al., the atypical frontoparietal synaptic gain was verified to mediate the generations of P300s in patients with psychtoic disorder when comapred to their unaffected relatives (Diez et al., 2017). Previous studies have proved the great importance of coupled top-down flows of the brain in regulating P300s (Li et al., 2015, 2016); meanwhile, the dysfunctions of multiple brain regions, such as dorsolateral PFC (DLPFC) and IPS, have also been claimed to be responsible for the cognitive deficits in SZs (Ohtani et al., 2014; Mouchlianitis et al., 2015).

In contrast to the functional specialization of related brain regions reported previously, the investigation of how the related functional integration extended on large-scale network adapts is now more urgent. We thereby hypothesized that the P300 deficits observed in SZs are attributed to the dysfunctioned functional integration (i.e., disconnectivity) of those coupled brain regions. Thereafter, in this work, we mainly concentrated on the extrinsic (between-sources) connectivity to investigate the “disconnectivity hypothesis” subserving the cognitive deficits in patients. And the visual oddball experiments and EEG-based DCM are simultaneously adopted to infer the deficits of cognitions induced by the dysfunctioned dynamics of coupled brain areas and how the brain adapts to promise the limited cognitions in SZs.

## Material and methods

### Participants

This study was carried out in accordance with the recommendations of Ethics Committee of Peking University Sixth Hospital. The protocol was approved by the Ethics Committee of Peking University Sixth Hospital. All subjects gave written informed consent in accordance with the Declaration of Helsinki. Prior to our experiments, all participants had been told about our experimental procedures; meanwhile, they were also required to read the written informed consent and then signed their name on it. Twenty-four SZs (14 females, age 33.63 ± 8.36) were recruited to take part in our current P300 experiments, as well as 24 HCs (8 females, age 29.88 ± 6.45) without personal or family history of psychiatric or neurological disease. All subjects had the normal or correct-to-normal vision. Meanwhile, all patients were interviewed by an experienced clinician to confirm their clinical diagnosis, as well as the Brief Psychiatric Rating Scale (BPRS).

### Experiment design

**Figure 1:**
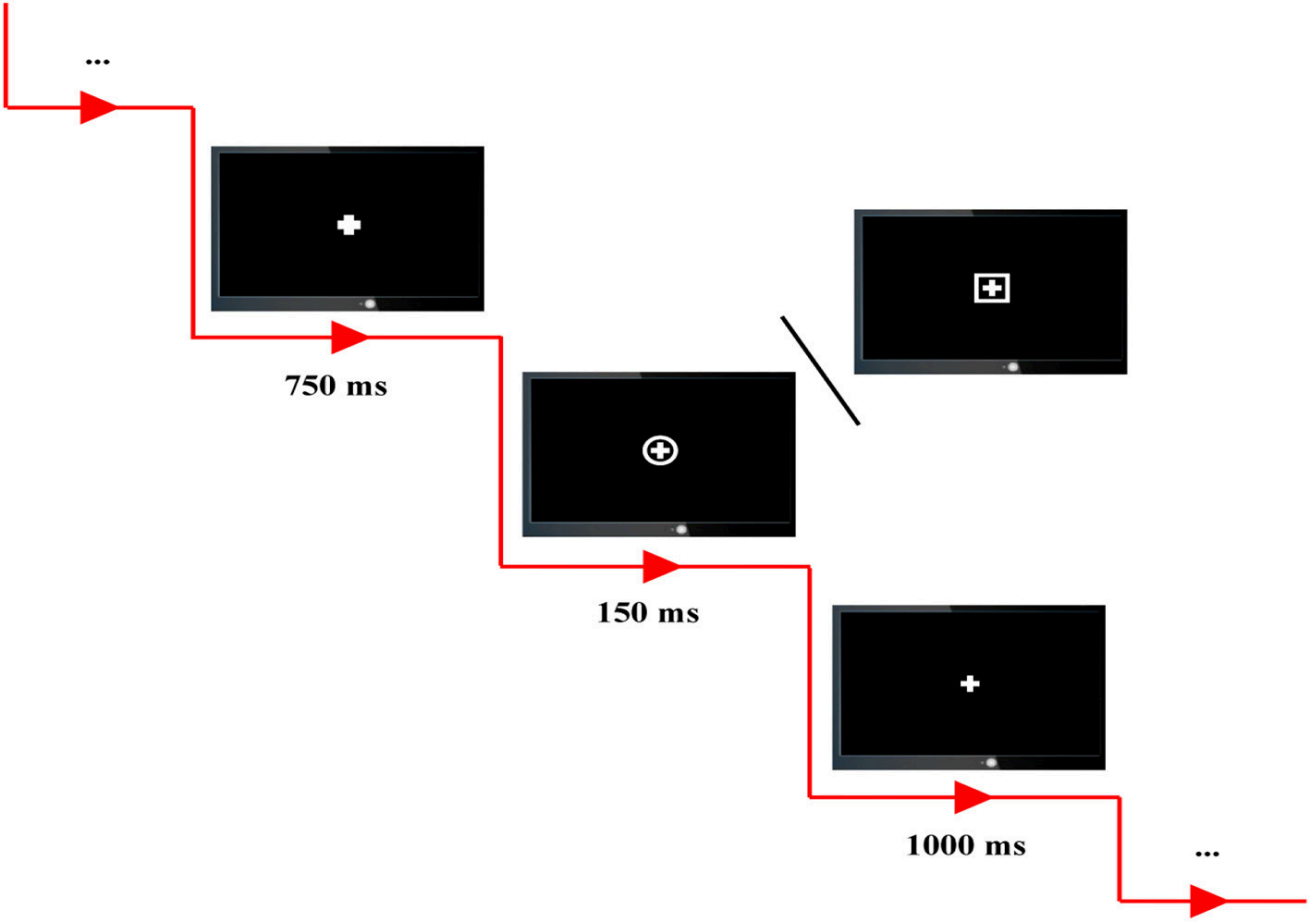
The experimental protocol. Combination of cross and square is defined as standard stimulus, and combination of cross and circle is defined as target stimulus. In each P300 trial, a 750-ms cue, 150-ms stimulus, and 1,000-ms rest are included.

All subjects were required to seat comfortably, to stay relaxed, and to avoid from blanking their eyes and moving their body during the whole experiments. Four runs of P300 tasks were included, and between each two neighboring runs, the subjects were given a 4-min break. In our experiments, the standard stimulus is defined as the combination of cross and square, and the combination of cross and circle is regarded as the target stimulus (Figure 1).

A total of 100 trials, 80 standards and 20 targets, were included in each P300 run and randomly presented on the computer screen. In each P300 trial, all subjects were instructed to fixate on the center of computer screen, and a 750-ms cue with the bold cross on the center of computer screen was firstly presented to warn the subjects to concentrate their attention on the screen without blanking their eyes and also to inform them that a standard/target stimulus would soon appear. Subsequently, a target or standard stimulus was presented with the time period of 150-ms; and the subjects were required to correctly press the corresponding key (i.e., “1” key in a standard keyboard) as quick as possible when the target stimuli were presented. After a 1,000-ms break represented by thin cross presented on the center of screen, the next trial was then initiated.

### Data recording

The task EEG datasets were recorded using the Symtop amplifier (Symtop Instrument, Beijing, China) with 16 Ag/AgCl electrodes (Fp1, Fp2, F3, F4, C3, C4, P3, P4, O1, O2, F7, F8, T3, T4, T5, T6) in compliance with the 10–20 international system. Electrode AFz served as the reference, and the EEG signals were sampled at 1,000 Hz with the online bandpass filter of 0.5–45 Hz. During the whole experiments, the impedances for all electrodes were consistently and consecutively kept below 5 KΩ.

### Data analyses

Using Matlab 2014a (The MathWorks Inc. USA), the EEG datasets were pre-processed to exclude the artifacts contained. The related procedures consisted of averaging-referencing, 0.5-to-30-Hz offline bandpass filtering, trial-segmenting starting 200 ms before and ending 800 ms after stimulus onset (0 ms represents the target stimulus onsets), and 200-ms baseline correcting. Thereafter, to automatically exclude those obvious artifacts with high amplitudes, a threshold of 100 μv was used to remove those P300 trials with their largest absolute value of amplitudes at any time point of any electrode over 100 μv. In addition, after the above procedures, the visual inspection of artifacts were further applied to remove the remaining artifacts, and this required the experimenters to visually check each remaining P300 trial of each subject. And if there existed some time points that had the highest amplitude over three times than that of any other time points in the same trial, then this trial would be also excluded from following analysis. Fortunately, no trials were excluded in this process. After this procedure, the numbers of remaining P300 trials were 49 ± 11.96 for HCs and 46.24 ± 18.51 for SZs. Finally, these artifact-free trials were trial-averaged with the final averaged trial being 2-rate down-sampled for each subject.

### P300 amplitude

The P300 is elicited by the target stimuli presented in classical oddball paradigm, and shows its positively largest peak at approximately 300 ms after the target onset and prominently over the parietal scalp regions (Sutton et al., 1965; Polich, 2007). Accordingly, in our present study, within the time interval of [300, 500] ms, we extracted the amplitude of the highest peak point on four electrodes (C3, C4, P3, and P4) that locate over the scalp parietal, and regarded the four amplitudes as the corresponding P300 amplitudes for each subject on the four electrodes. In addition, to statistically uncover the possible differences of P300 amplitudes between SZs and HCs, the independent *t*-test was adopted to perform the comparison of amplitude on the four electrodes between the two groups.

### Dynamic causal modeling

**Figure 2:**
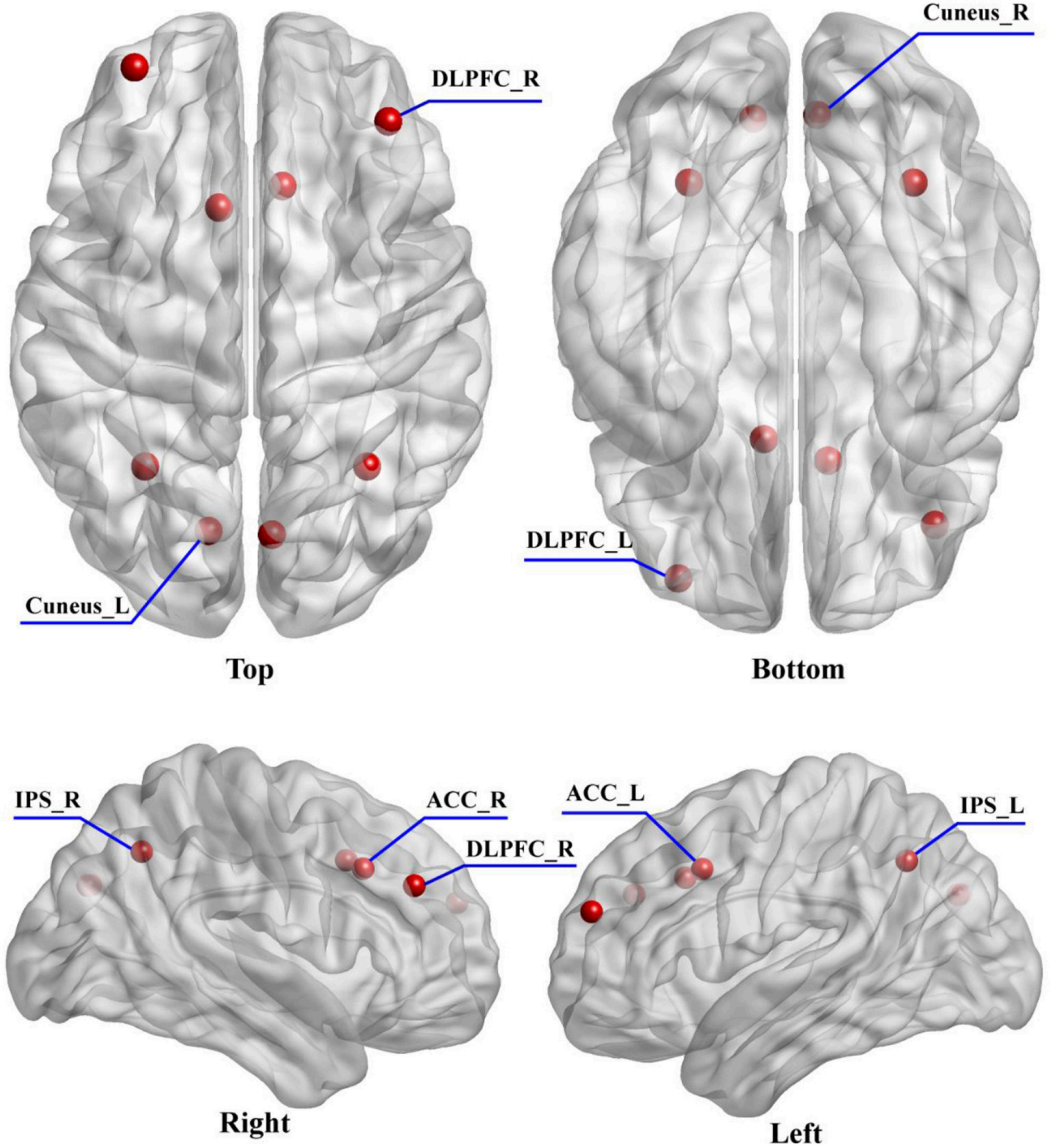
The distribution of selected eight DCM nodes drawn on the brain. The eight regions include the left (−33 54 24) and right (39 39 30) DLPFC, left (−9 15 39) and right (9 21 36) ACC, left (−30 −57 42) and right (33 −57 42) IPS, and left (−12 −75 30) and right (6 −76 30) cuneus.

In order to obtain the plausible spatial coordinates of the P300 generators, prior to DCM study, a literature review of related studies was performed (Clark et al., 2000; Stevens et al., 2000; Bledowski et al., 2004; Brázdil et al., 2007; Musso et al., 2011). In this study, we intended to investigate how the dysfunctions of functional integration (i.e., “disconnectivity”) occurred and how the brain adapts to promise the related information processing. Therefore, from posterior (visual cortex) to anterior lobes (frontal cortex), the six brain regions locating at bilateral visual, parietal, and prefrontal cortices were marked. Meanwhile, the crucial importance of ACC in the generations of P300s (Brázdil et al., 2007) and its dysfunction leading to the cognitive deficits in SZs (Wang et al., 2004) have been widely reported, which inspired us to take ACC into consideration. Therefore, to keep the model space as simple as possible, in our present study, the final selection of DCM nodes comprised eight brain regions including bilateral cuneus, IPS, ACC and DLPFC; and the bilateral cuneus were selected as the initial processing step (i.e., input region). The corresponding prior Montreal Neurological Institute (MNI) coordinates of eight DCM nodes were obtained from Brázdil et al. (2007) and the related distribution were depicted in Figure 2.

**Figure 3:**
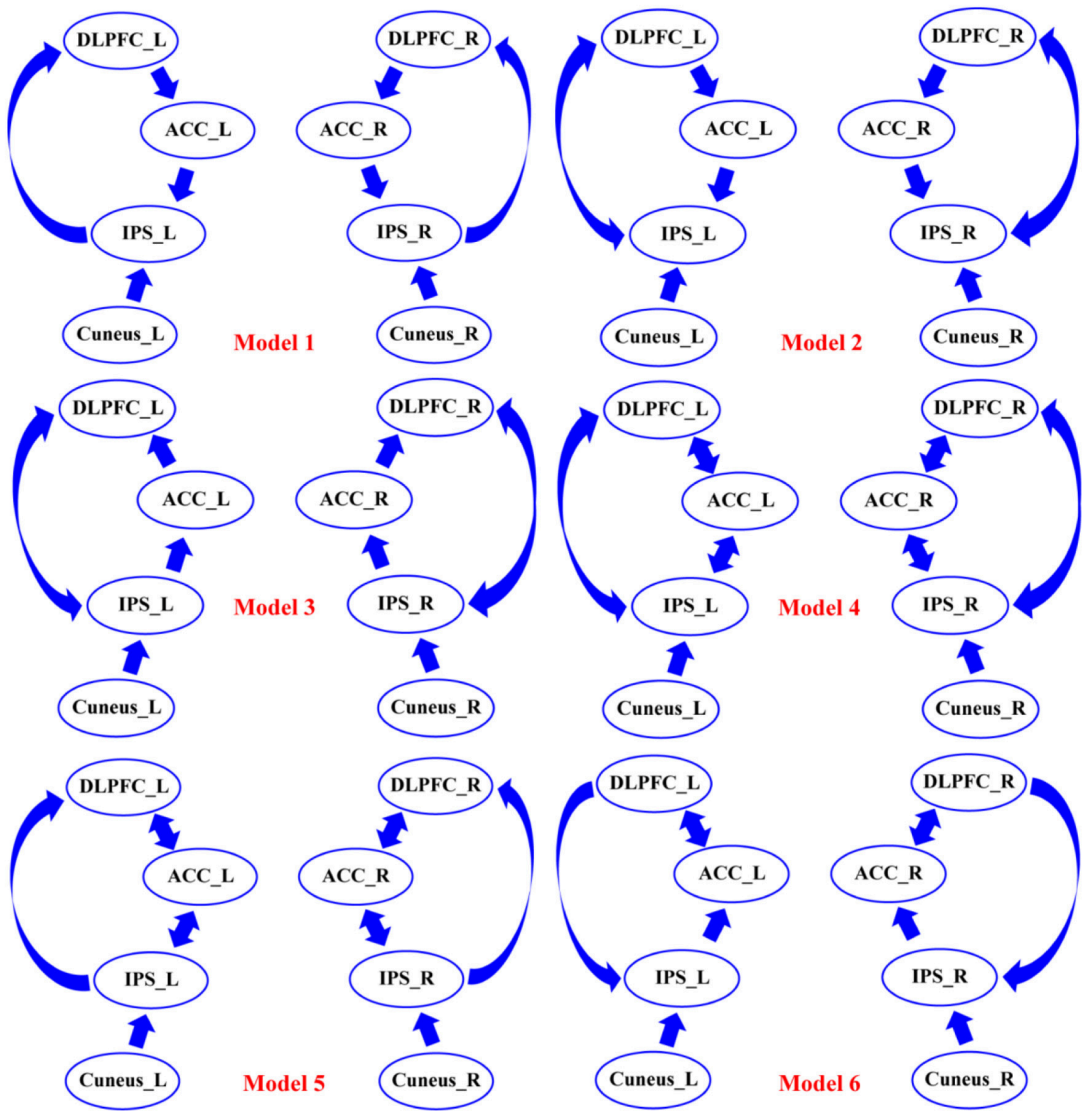
The six assumptions of DCM connectivity among the eight DCM nodes. Among six models, the possible forward and backward flows were defined based on the existing knowledge of P300. DLPFC, dorsolateral prefrontal cortex; ACC, anterior cingulate cortex; IPS, intraparietal sulcus; L, left; R, right.

Thereafter, all of the condition-specific grand-averaged P300 data were converted into the Statistical Parametric Mapping (SPM) format separately for SZs and HCs. In the present study, the sources of P300 were modeled as single Equivalent Current Dipoles (ECD) under the bilateral symmetry assumptions (Kiebel et al., 2006). Then, the Canonical Microcircuit neural mass model (Bastos et al., 2012) was adopted, and in this model, each neural source comprises four cell populations including the superficial and deep pyramidal cells, inhibitory interneurons, and spiny stellate cells. The Boundary Elements Model (BEM) (Fuchs et al., 2001) was then adopted as approximation to the brain, cerebrospinal fluid, and skull and scalp surfaces; meanwhile, a structural MRI head model was used for the co-registration of 16-channels positions. Then, Bayesian Model Selection (BMS) (Penny et al., 2004) finds the DCM model with the largest log-evidence among those models considered, which can explain the scalp EEGs on the 16 channels well. Importantly, before testing for “disconnectivity hypothesis” in SZs, we established the best models that could explain the task effects across groups. In our current work, we adopted the BMS to identify which model could better explain our data and the corresponding results. As the DCM requires our prior knowledge about the investigated issue, based on the eight activated sources and the P300 related networks proposed in previous studies (Brázdil et al., 2007; Chen et al., 2014; Li et al., 2016), we pre-defined six possible connectivity models that were mainly concentrated on probing how the flow of top-down or bottom-up works in the elicitations of P300s, as well as the crucial role of ACC in explaining the cognitive deficits in SZs (Figure 3). On the one hand, in our present study, for either top-down or bottom-up flow, we hypothesized two distinct directed pathways; in detail, for top-down, the DLPFC-to-ACC-to-IPS, and DLPFC-to-IPS were hypothesized, and for bottom-up, the IPS-to-ACC-to-DLPFC and IPS-to-DLPFC were assumed in Figure 3. Therefore, in each subfigure of Figure 3, either top-down or bottom-up or their combinations could be found. On the other hand, based on the hypothesized distinct pathways of top-down/bottom-up, we further intended to prove if the ACC was crucial in the related information processing (i.e., relay station) by focusing on the differences between the concerned models, i.e., models 4 and 5, models 1 and 2, and models 3 and 4, etc. Meanwhile, by comparing the models 4, 5, and 6, we simultaneously investigated the disconnectivity of ACC in the brain to probe if its disconnectivity could account for the cognitive deficits in SZs.

Based on all of six pre-defined DCM models of extrinsic connectivity (i.e., connectivity between sources), SPM8.0 (available at website: http://www.fil.ion.ucl.ac.uk/spm) (Friston et al., 1994) was used to perform our DCM analysis at the group level (Ranlund et al., 2016). After the winning models for SZs and HCs were selected, based on the winning models, we analyzed the flow differences between two groups, and speculated the possible explanations that can account for the cognitive deficits in SZs.

## Results

**Figure 4:**
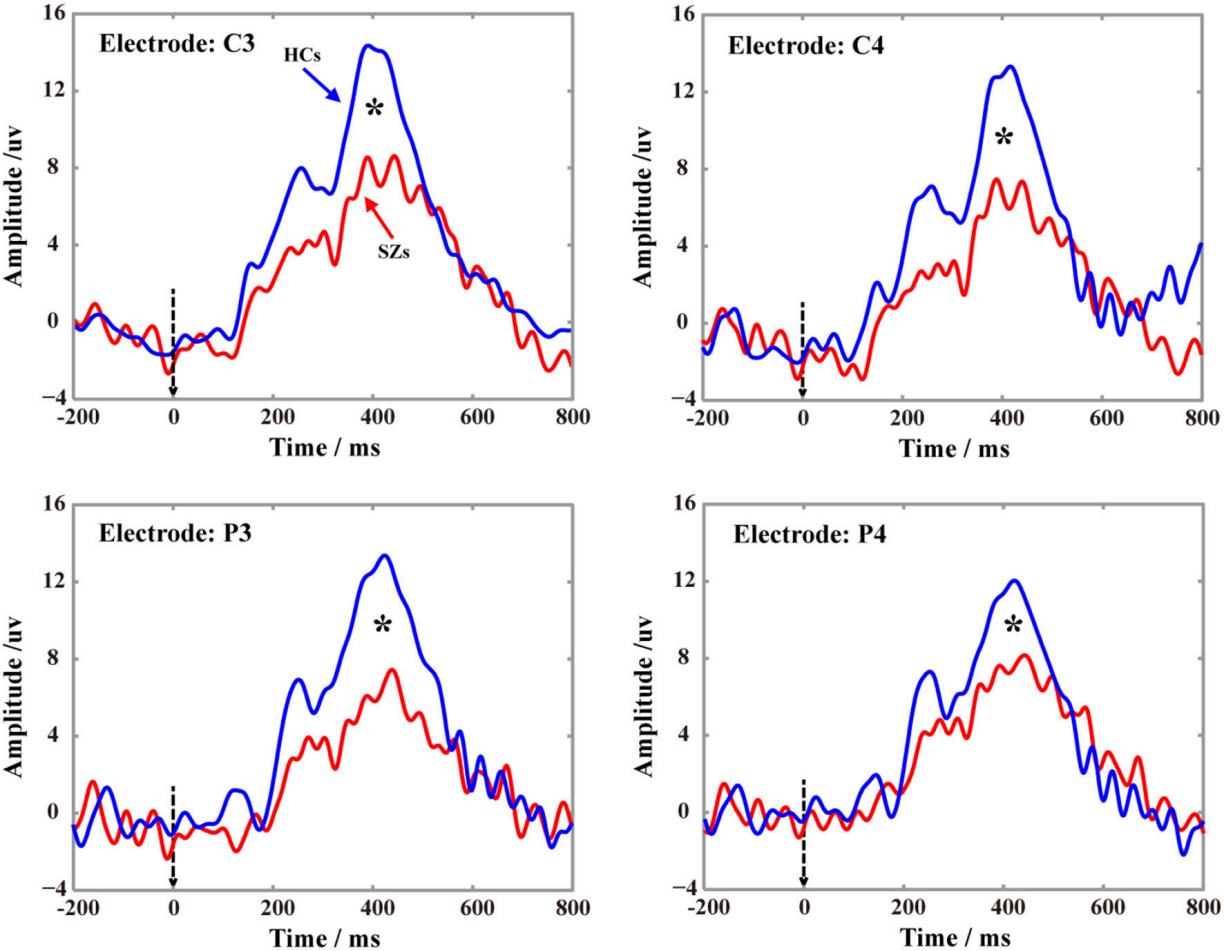
Grand-averaged P300 waveforms for SZs and HCs on four electrodes (C3, C4, P3, and P4) recorded. In each subfigure, the blue solid line denotes the P300 of HCs, the red solid line denotes the P300 of SZs, and the black star indicates the significance level of P300 peak (*p* < 0.05) between HCs and SZs.

The P300 potentials in Figure 4 demonstrate the P300s are elicited on four electrodes (C3, C4, P3, and P4) for both SZs and HCs during performing the P300 tasks, and the relatively low P300 amplitudes were consistently found in SZs on four electrodes. Meanwhile, the statistics of P300 amplitudes between SZs and HCs (*p* < 0.05) further showed the significant reductions of P300 amplitudes in SZs, compared to that in HCs.

**Figure 5:**
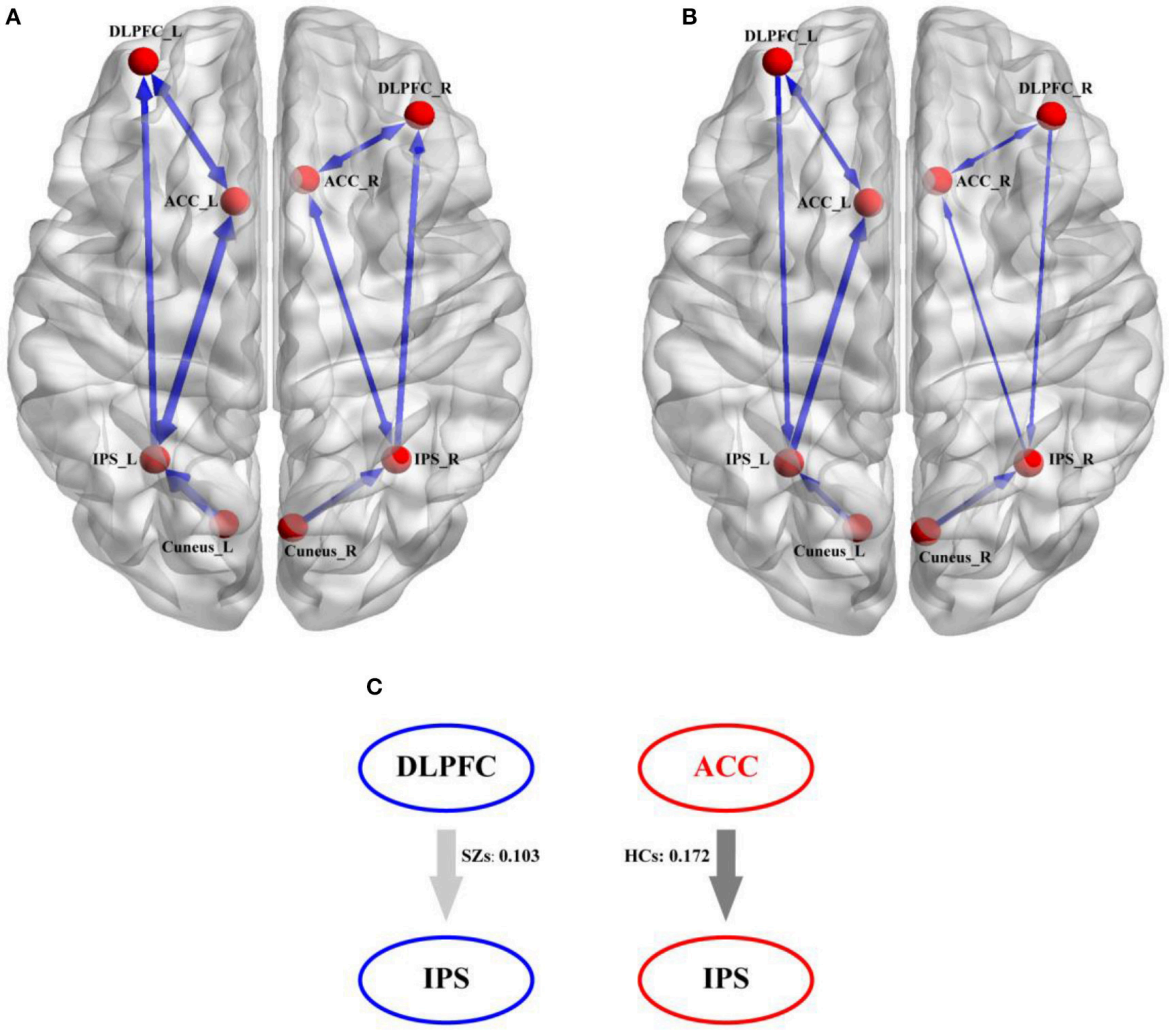
The directed information exchanges in HCs and SZs derived from their own winning DCM model. **(A)** The winning DCM model in HCs, **(B)** the winning DCM model in SZs, and **(C)** the strength differences of direct information flows between flow from ACC to IPS in HCs and flow from DLPFC to IPS in SZs. In **(A,B)**, the blue solid arrows denote the direction of the information flow between two nodes and the thicker blue lines denote the corresponding stronger information exchange.

The distinct winning models were selected by the BMS, model 5 for HCs (Figure 5A) and model 6 for SZs (Figure 5B) in Figure 5. In Figures 5A,B, the distinct thicknesses of sold lines with blue arrows denote the actual strength of information exchange between two correlated nodes, and the thicker lines represent the stronger flows. From Figures 5A,B, the denser linkages of ACC with other regions, such as DLPFC and IPS, could be clearly observed. Meanwhile, comparing to that in HCs, for SZs, no flow from ACC to IPS was found, which was replaced by the directed flow from DLPFC to IPS; and the directed flow from IPS to DLPFC could only be observed in HCs. This compensative connection from DLPFC to IPS may serve the similar function as that accomplished by flow from ACC to IPS. Therefore, to further probe the possible difference of information propagation between flow from ACC to IPS in HCs and flow from DLPFC to IPS in SZs, Figure 5C illustrates the mean flow strengths for the two connections between the two groups (i.e., 0.103 for SZs and 0.172 for HCs), and the independent *t*-test further revealed the significantly (*p* < 0.05) stronger strength of directed flow from ACC to IPS in HCs than that of directed flow from DLPFC to IPS in SZs.

## Discussion

In the present study, the DCM was adopted to investigate the potential mechanism (“disconnectivity hypothesis”) that accounts for the variations in processing the P300 information between two groups, as well as the related cognitive deficits in SZs. We firstly statistically compared the differences of P300 amplitudes between two groups on four electrodes, which were shown in Figure 4, and further illustrated the similar findings reported by previous studies that though both HCs and SZs could be found the P300 potentials, SZs still showed the significant deficits of P300 (i.e., the smaller P300 amplitudes in our present study).

Based on the selected winning models in Figure 5 (i.e., model 5 for HCs and model 6 for SZs), we assumed that the target related information is initially inputted into bilateral cuneus; after the integration, the information will be subsequently transferred to the IPS and ACC. In ACC, a series of consecutive plans (i.e., regulating DLPFC to do the decision-making, controlling IPS to respond to target stimuli) will be formulated. After being coded in DLPFC, these commands will be inversely propagated to the IPS and the P300s are thereby elicited. In fact, the efficient architecture in the brain promises the higher P300 amplitudes. Once the disconnections of brain architecture happen, the P300 deficits occur (Yamaguchi and Knight, 1991; Daffner et al., 2003), which was consistently illustrated in Figure 4. In the present study, comparing to HCs, the directed information flow from DLPFC to ACC and missing flow from ACC to IPS could be found for SZs, we thereby assumed that the disconnection from ACC to IPS fails to transfer the commands to the task-related regions to further control the neuronal activity, which led to the P300 deficits in SZs.

Comparing to HCs, Figure 5 further provided the strength differences on the directed flows from ACC to IPS, from DLPFC to IPS, and from IPS to DLPFC. In fact, ACC is related to the distinct processes, such as learning and selecting high-level plans, control execution, and action selection (Holroyd and Yeung, 2012); while DLPFC is usually responsible for the information encoding and decision processing (Duncan, 2001; Barraclough et al., 2004). The key difference between them is that ACC instigates the sequential switches to achieve a higher-level goal (i.e., getting in the car, driving to the market, getting the groceries); whereas DLPFC only implements the task at hand (i.e., driving to the market) (Holroyd and Yeung, 2012). In the present work, in processing the progress of elicitations of P300, ACC firstly instructs DLPFC to code the information to the command, and then transfers the command back to IPS to “tell” IPS to accomplish the required responses. For SZs, the loss of directed flow from ACC to IPS makes it impossible to transfer the command directly to IPS. To make up this disconnection, the brain recruits another backward pathway, which may be attributed to the compensatory plasticity of the brain (Rauschecker, 1995). Similar to the blind patients who compensated for their lack of vision by better developing their remaining senses (Kupers and Ptito, 2014), patients with Parkinson's disease recruited the cerebello-thalamo-cortical loop to compensate for their dysfunctional basal ganglia circuits (Palmer et al., 2010; Schroll et al., 2014). Meanwhile, Basten et al. further proved the increased DLPFC activation in anxious individuals that was thought to be an attempt to compensate for the suboptimal connectivity within the cortical network (Basten et al., 2011), as well as in SZs (Cieslik et al., 2015). In our present study, comparing to HCs, though no directed flow from ACC to IPS was found for SZs, another compensatory flow originating from DLPFC and ending at IPS could be found in Figure 5B. Although the weaker coupling strength of DLPFC to IPS in SZs was revealed by Figure 5C in our present work than that of ACC to IPS in HCs, we still assumed that this DLPFC to IPS pathway might be a compensatory of the directed flow from ACC to IPS, which guaranteed SZs to accomplish the required P300 tasks, though the corresponding amplitude was lower and latency was longer.

In conclusion, SZs showed the disconnectivity in their brain architecture during the related cognitive process. For SZs and HCs, the different winning models were selected, which consistently proved that ACC was crucial in P300 elicitation. And comparing to HCs, the directed flow from ACC to IPS was compensated by the flow from DLPFC to IPS to guarantee SZs' accomplishing the P300 experiments. Our present findings confirmed the present of top-down disconnectivity in schizophrenia that subserves the cognitive deficits in SZs; meanwhile, the compensate mechanism is simultaneously verified to compensate for the atypical cognitive information processing in SZs, which guarantees the SZs to accomplish the cognitive processes to some extend and further deepens our understanding of the cognitive deficits in schizophrenia.

Although both Granger Causal Analysis (GCA) and DCM approaches can be used to estimate the directed influences between two concerned variables, the GCA is one of the data-driven methods, which is based on the mathematical characterizations of given variables. In contrast, the DCM is one of the model-driven approaches that consider the physiological models, its biological basis is that one neuronal system exerts its effects on another. Recently, due to the biophysical derivations, the DCM thereby was adopted in our present study to probe the potential mechanism that accounts for the cognitive deficits in SZs from the aspect of networks. One possible limitation of present study might be that the relatively low-density distribution of scalp electrodes was used during the EEG data recording, which may not provide the enough spatial information. In our future study, the more electrodes will be considered for the related clinical studies.

## Author contributions

PX, JW, and WD conceived of and designed the experiments. JW performed the experiments. FL, YuJ, WP, LS, and YiJ analyzed the dataset. FL and PX wrote the manuscript. YS, YZ, and DY provided some useful suggestions in manuscript writing.

### Conflict of interest statement

The authors declare that the research was conducted in the absence of any commercial or financial relationships that could be construed as a potential conflict of interest.
